# H3 Lysine 4 Methylation Is Required for Full Activation of Genes Involved in α-Ketoglutarate Availability in the Nucleus of Yeast Cells after Diauxic Shift

**DOI:** 10.3390/metabo13040507

**Published:** 2023-03-31

**Authors:** Elena Di Nisio, Svetlana Danovska, Livia Condemi, Angela Cirigliano, Teresa Rinaldi, Valerio Licursi, Rodolfo Negri

**Affiliations:** 1 Department of Biology and Biotechnologies “C. Darwin”, Sapienza University of Rome, 00185 Rome, Italy; 2 The Center for Genomic Regulation (CRG) and the Barcelona Institute of Science and Technology (BIST), 08001 Barcelona, Spain; 3 Institute of Molecular Biology and Pathology (IBPM), National Research Council (CNR) of Italy, 00185 Rome, Italy

**Keywords:** H3K4 tri-methylation, diauxic shift, transcriptional regulation

## Abstract

We show that in *S. cerevisiae* the metabolic diauxic shift is associated with a H3 lysine 4 tri-methylation (H3K4me3) increase which involves a significant fraction of transcriptionally induced genes which are required for the metabolic changes, suggesting a role for histone methylation in their transcriptional regulation. We show that histone H3K4me3 around the start site correlates with transcriptional induction in some of these genes. Among the methylation-induced genes are *IDP2* and *ODC1*, which regulate the nuclear availability of α-ketoglutarate, which, as a cofactor for Jhd2 demethylase, regulates H3K4 tri-methylation. We propose that this feedback circuit could be used to regulate the nuclear α-ketoglutarate pool concentration. We also show that yeast cells adapt to the absence of Jhd2 by decreasing Set1 methylation activity.

## 1. Introduction

Chromatin structure governs several aspects of cell metabolism. Histone N-terminal tails are subjected to several covalent modifications which form a sophisticated combinatory code interpreted by a plethora of regulatory protein complexes [1,2]. Among the various modifications, Lysine (K) methylation is particularly interesting, due to its widespread roles in transcriptional regulation, DNA repair and epigenetic inheritance. In *S. cerevisiae*, three lysine methyl transferases, Set1, Set2 and Dot1, catalyze histone mono-, di- or tri-methylation at K4, K36 and K79, respectively. These epigenetic marks, which are absolutely conserved among eukaryotes, have been associated with actively transcribed loci [3], although their roles in controlling transcription efficiency may be distinct and strongly context-dependent [4]. H3K4 tri-methylation is enriched at the promoters and 5′ portions of actively transcribed open reading frames (ORF) in both yeast and higher eukaryotes [5] and seems to play multiple, variable and sometime conflicting roles in transcription [6,7,8,9,10]. 

Two families of histone demethylating enzymes (HDMs) have been identified in eukaryotes: the lysine-specific demethylase 1 (LSD1) family and the Jmjc-domain-containing family [11]. Jumonji C domain-containing HDMs (JHDMs), 5 members in *S. cerevisiae* and at least 28 members in *H. sapiens*, are Fe^2+^ and α-ketoglutarate-dependent hydroxylases, and their reported substrate residues include H3K4, H3K9, H3K27, and H3K36 at all methylation states. The JHDM Jhd2 (encoded by YJR119c) purified from budding yeast specifically removes H3K4 di- and tri-methylation [12,13]. Its deletion does not show dramatic phenotypes, leading to a modest increase of H3K4me3 in bulk chromatin of exponential growing cells [12]. This observation suggests that its action could be strictly regulated and required mainly in specific physiological states of the cell. Indeed, important regulative roles of Jhd2 have been demonstrated during sporogenesis [14] and pseudohyphal differentiation [15] and in mitotic rRNA condensation [16]. Moreover Radman-Livaja et al. [17] have previously shown that active demethylation is required to erase H3K4me3 waves associated with wide transcriptional reprogramming in vivo. In budding yeast, a major regulative transition is represented by the diauxic shift (DS), which is triggered by glucose limitation and characterized by activation of glycogen catabolism and slowing down of replication rate. This involves a global reprogramming of the cell transcriptome characterized by a general repression of genes expressed during exponential growth and activation of a selected set of genes [18,19]. The aim of this work was to elucidate the role of H3K4 methylation level in this transcriptional reprogramming. We show here that it is indeed in part associated with a H3K4 trimethylation increase and that the methylation state regulates some of DS induced genes. We focused our analysis in particular on *IDP2* and *ODC1*, two genes which control the availability of α-ketoglutarate in the nucleus [20,21] and may therefore influence the activity of Jhd2 itself.

## 2. Materials and Methods

### 2.1. Yeast Growth Condition

All yeast strains were grown in YP medium supplemented with 2% glucose (YPD) in 0.5 L flasks kept at 30 °C in agitation on orbital oscillators. Overnight cultures of *S. cerevisiae* strains (Table 1) were used to inoculate 100 mL of fresh YPD medium at 0.1 A_600_. Cells were grown to 2 × 10^7^ (exponential growth—EG) or to 4 × 10^8^ (diauxic cells—DS) cells/mL.

### 2.2. Chromatin Immuno-Precipitation on DNA Arrays (ChIP on Chip)

BY4741 cultures were fixed by adding 1% formaldehyde for 15 min at room temperature. Fixation was stopped by adding 340 mM glycine and incubating for 5 min. Fixed cells were harvested by centrifugation and washed twice with TBS. Microarray slides were kindly provided by ENS transcriptomic platform (Paris, France) and ChIP on chip procedures were described in [22]. Briefly, ChIP samples were incubated with anti-H3K4me3 (Cell Signaling, Danvers, MA, USA, rabbit polyclonal, 1:1000), coupled to 20 µL of proteinA-agarose, previously equilibrated in Wash buffer (10 mM Tris-HCl pH8, 0.25 M LiCl, 0.5% NP-40, 0.5% sodium deoxycholate, 1 mM EDTA) and recovered by centrifugation. Microarrays were scanned using a double-laser scanner (Packard BioChip Technologies, Billerica, MA, USA) and fluorescence ratios were determined with GenePix Pro software version 5.1 (Axon Instruments, Scottsdale, AZ, USA). Local background signal was subtracted from the intensity of the spots signal. The arrays were within-normalized using the print-tip loess normalization. Log2(IP/WCE) was calculated for each DNA sequence. Only loci with a significant signal in both experiments were considered. The IP/Input ratios obtained in the two experimental points were ordered in percentile rank and compared in order to find differences in rank between them. The genomic regions showing a remarkable increase, average of over 20 percentiles in their rank between DS and exponential cells were collected in a list containing 86 ORF and 85 intergenic regions.

### 2.3. Western Blot Analysis

Yeast extracts for western blot analysis were prepared using standard glass bead disruption into a buffer A (50 mM Tris HCl at pH7.5, 2 M Sucrose, 5 mM MgCl2, 1mM DTT, Complete protease inhibitor cocktail from Roche, Basel, Switzerland), 45 min at 4 °C. Lysed cells were centrifuged at 3100 rpm for 15 min at 4 °C and pellets were resuspended in buffer B (20 mM HEPES pH 7.5, 1.5 mM MgCl2, 0.5 M NaCl, 0.2 mM EDTA, 20% Glycerol, 1% Triton X-100, 1 mM DTT, Complete protease inhibitor, Roche, Basel, Switzerland).

Yeast extracts were loaded on 15% (histones) or 10% SDS-PAGE polyacrylamide gels and after the run transferred on nitrocellulose membranes (Whatman, Little Chalfont, UK) by TransBlot method (Bio-Rad, Hercules, CA, USA) in 25 mM Tris, 192 mM Glycine, 29% Methanol, 1 h, 100 V at 4 °C.

Membranes were hybridized with the following antibodies: H3 (Active Motif, Carlsbad, CA, USA, rabbit polyclonal 1:1000); anti-tri-methyl H3K4me3 (Cell Signaling, rabbit polyclonal, 1:1000); anti-di-methyl H3K4me2 (Active Motif, rabbit monoclonal, 1:1000); TAP-tag Antibody (GeneScript, Rijswijk, The Netherlands, rabbit polyclonal, 1:1000); anti-Set1 (Santa Cruz Biotechnology, Dallas, TX, USA sc-101858, mouse monoclonal 1:1000). Chemiluminescence signals intensity ratios were quantified by chemiluminescence imaging with the ChemiDoc™ XRS (Bio-Rad).

### 2.4. Chromatin Immunoprecipitation (ChIP)

Yeast cells grown as described above were cross- linked with 1% formaldehyde for 30 min before chromatin was extracted. The chromatin was sonicated (20 cycles, 60 s on/off, high setting) to yield an average DNA fragment of 500 bp. H3K4me3 (Cell Signaling, rabbit polyclonal, 1:1000) were coupled to 20 µL of proteinA-agarose, previously equilibrated in Wash buffer (10 mM Tris-HCl pH8, 0.25 M LiCl, 0.5% NP-40, 0.5% sodium deoxycholate, 1 mM EDTA). After reversal of the crosslinking and DNA purification, the immuno-precipitated and input DNA was analyzed by quantitative real-time PCR. 

An amplicon designed in the coding region of *ZRT1*, a gene not regulated by DS, which showed no H3K4me enrichment between DS and exponential cells (ChIP on chip data) has been used as endogenous calibrator.

### 2.5. Real Time RT-PCR

One microgram of RNA from samples was reversed transcribed using SuperScript™ III RT (Invitrogen, Waltham, MA, USA, Cat. Num. 18080-044 200 units/µL) with 1 µL of oligo(dT)20 (50 µM) according to the manufacturer protocol. The cDNA served as template for subsequent Real Time RT-PCR reactions that were set up in duplicate for each sample using the Sensimix SYBR Real Time Mix (Bioline, London, UK, Cat. Num. QT-606) using an Applied Biosystems Prism 7300 Sequence Detector (Thermo Fisher Scientific, Waltham, MA, USA). The reaction mixtures were kept at 95 °C for 10 min, followed by 40 cycles at 95 °C for 15 s and 60 °C for 1 min. The level of transcripts was evaluated by primers reported in Table S1.

Fluorescence output was analyzed using Sequence Detection Software, version 1.2 (Thermo Fisher Scientific, Waltham, MA, USA). Relative quantification was carried out with the 2^−ΔΔCt^ method, using the abundance of Taf10 (a gene not significantly regulated through DS) or ACT1 as endogenous house-keeping control. Data were statistically analyzed by Student’s *t*-test.

### 2.6. Statistical Analysis

Data were analyzed using R version 4.2 and Microsoft Excel version 16. Statistical analysis was conducted using unpaired two-sided Student’s *t*-test.

## 3. Results

### 3.1. Expression of Jhd2 and Set1 in Different Phases of Yeast Growht

Both Set1 and Jhd2, the enzymes which control the methylation state of H3K4, were previously shown to be expressed during Diauxic Shift (DS) and early stationary phase (ref. [18,19] and Figure S1A) suggesting a specific role for their histone modification action in transcriptomic reprogramming. In particular: *JHD2* mRNA increases prior to DS and remains very abundant until post-diauxic phase, while *SET1* mRNA shows a bimodal accumulation, peaking in exponential phase and at the end of DS (Figure S1). This suggests a possible prominent role of Set1 in regulating genes important for DS onset and of Jhd2 for balancing this action. We therefore asked how was *JHD2* regulated at the exit of DS, when fresh glucose rich medium is given back to the cells. The experiment, reported in Figure S1B, confirms that *JHD2* mRNA is present during DS (contrary to ribosomal proteins (RP) mRNAs which are completely absent) but is strongly induced at 30 min from DS exit, when fresh medium with glucose is added to the cell culture. Western blots performed with the Tap-tagged Jhd2 strain shows that the protein follows the same trend of the mRNA, being present in DS and further accumulated immediately after addition of fresh medium (Figure S1C). These results indicate that the Jhd2 demethylase could have a specific role at DS entry and exit.

### 3.2. H3K4 Tri-Methylation Level during DS

**Figure 1:**
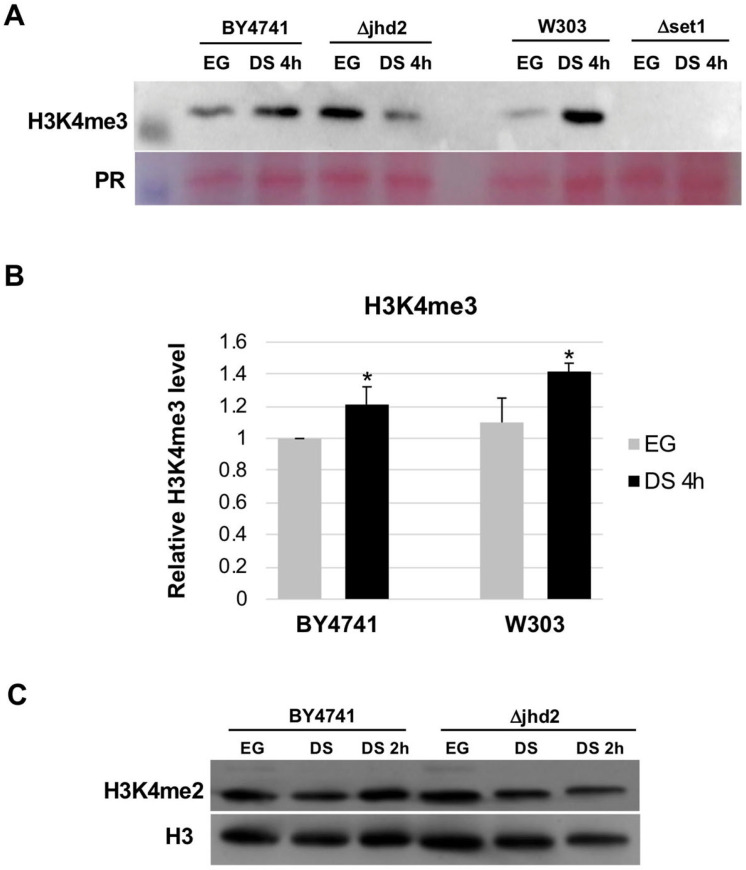
(**A**) Western blot showing H3K4me3 level in wild type (BY4741, W303) and mutant (ΔJHD2, ΔSET1) strains during exponential growth (EG) or 4 h after diauxic shift (DS). The phenol red stain of histones is reported as loading control. (**B**) Quantitation of H3K4me3 at EG and 4 h after DS in wild type strains. *n* = 3, standard deviation is indicated. * = *p* < 0.05 according to Student’s *t*-test. (**C**) Western blot showing the level of H3K4me2 during EG, DS and 2 h after DS in the BY4741 and ΔJHD2. The histone H3 is the endogenous calibrator.

The previous observations suggest that H3K4 methylation balance could be involved in the DS transition. We therefore tested H3K4 tri-methylation levels in bulk chromatin before and after DS. Cells from strains BY4741 and W303 were grown in YPD and samples were taken during exponential growth (EG) and two hours after DS (defined as the time at which growth plateau is reached). Total histones were acid-extracted and analyzed by western blot with anti-H3K4me2 and H3k4me3 antibodies. Figure 1A,B show that in both wild type strains analyzed (BY4741 and W303) there is an evident increase of H3K4 tri-methylation at 4 h after DS as compared with exponential growth. H3K4me2 level appears modestly increased too (Figure 1C). We also analyzed two BY4741-derived strains carrying deletions in *JHD2* and *SET1*, respectively. While the ΔSET1 obviously does not show any H3K4 tri-methylation, the result for the ΔJHD2 is quite unexpected: the level of H3K4 di- and tri-methylation tends to decrease in DS (Figure 1A,C). 

To identify which genomic regions show an increase of H3K4-trimethylation at DS we performed a global screening by ChIP on chip using microarray slides containing the whole repertoire of *S. cerevisiae* ORFs and intergenic sequences (see Section 2). We immuno-precipitated chromatin from exponential and diauxic shifted cells of the BY4741 wild type strain. IP and Input samples were amplified and labelled with Cy5 e Cy3 respectively, in order to test the H3K4me3 enrichment on ORFs and intergenic regions of the whole genome. The IP/Input ratios obtained in the two experimental points were compared to find differences in the enrichment between them. The genes showing an average increase of H3K4 tri-methylation at least 20 in percentile ranking between DS and EG either in the ORF or in the upstream intergenic sequence were selected in a list (Table S2) containing 86 ORF and 85 intergenic regions. This list (DS-hypermethylated) was compared with a list containing the genes significantly induced at least 3-fold during diauxic shift as compared to exponential phase (DS-induced, ref. [19]). We found a significant (*p* < 0.001) overlapping between the two lists (45 genes), 5-fold higher than expected by chance (9 genes). Several of these genes are involved in the change of metabolism imposed by DS. Notably, *IDP2* and *ODC1*, two genes which regulate the nuclear concentration of α-ketoglutarate, the Jhd2 demethylase cofactor, are among the 15 most tri-methylated genes (reported in Table 2) ranking 1 and 7, respectively. We found a much lower number of genomic loci showing an evident decrease of H3K4 tri-methylation (Table S2) containing genes repressed at DS.

**Figure 2:**
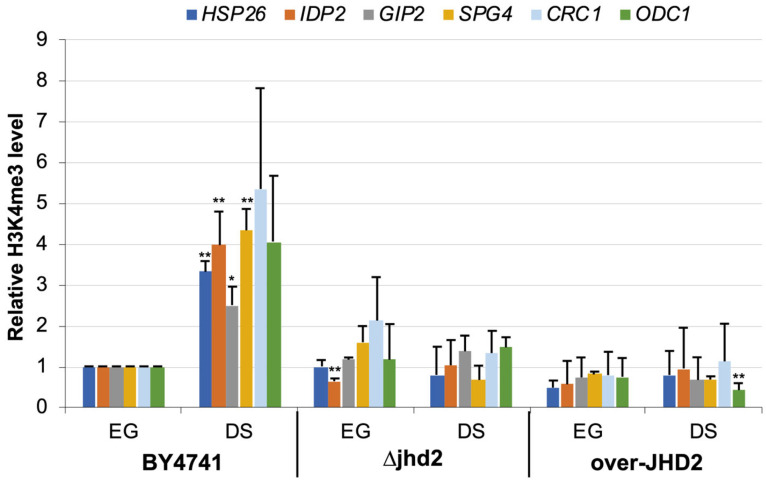
H3K4 trimethylation in the proximal ORF portion of HSP26, IDP2, GIP2, SPG4, CRC1 and ODC1 at EG and DS in the wild type strain BY4741; in the isogenic ΔJHD2 strain and in a strain overproducing Jhd2 (over-JHD2, see Table 1). * = *p* < 0.05; ** = *p* < 0.01 according to Student’s *t*-test.

Next, we validated some of the data by ChIP followed by real time quantitative PCR, using amplicons designed in the ORF and in the promoter of genes selected among those reported in Table 2. In all cases after DS the wild type BY4741 strain shows a significant increase (at least two-fold) of H3K4me3 in the proximal (within the first 500 bp) segment of the ORF (Figure 2).Strikingly, when we repeated the experiments with the strain deleted in *JHD2* we did not observe any significant increase in H3K4 trimethylation, exactly as observed for a strain carrying a multicopy plasmid over-expressing the Jhd2 demethylase (Figure 2). This paradoxical result is coherent with the previously shown general decrease of H3K4me3 level in bulk histones of the strain lacking the Jhd2 demethylase and suggests the existence of a mechanism of adaptation of the deleted strain to histone methylation imbalance (discussed below). We also tested HAS1, one of the few genes which showed a strong H3K4 trimethylation decrease in the ChIP on chip screening which confirmed to be extensively demethylated at DS in the WT strain but did not show significant difference in the ΔJHD2strain (Figure S2).

### 3.3. H3K4 Methylation Stimulates Gene Transcription Induction at DS

**Figure 3:**
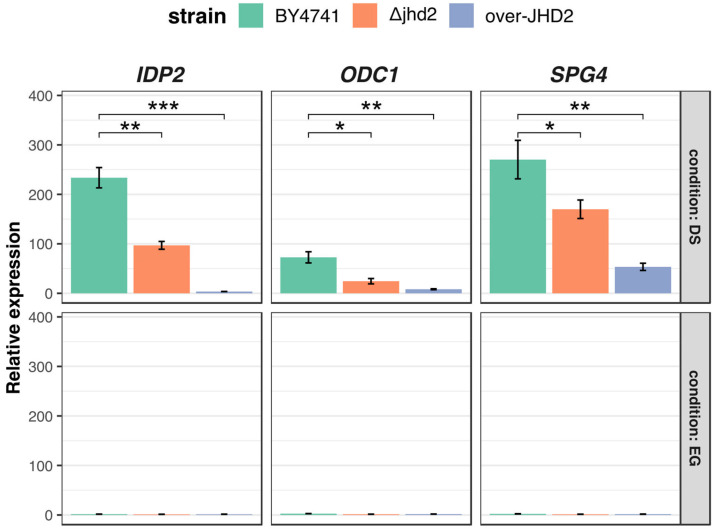
Real time RT-PCR quantitation of cDNA of IDP2, ODC1 and SPG4 at EG or DS for BY4741, ΔJHD2 and over-JHD2 strains. n = 3, standard deviation is indicated. * = *p* < 0.05; ** = *p* < 0.01; *** = *p* < 0.001 according to Student’s *t*-test.

In order to test the effect of H3K4 methylation on gene transcription induction at DS, we grew cells from the wild type BY4741 and from ΔJHD2, or Jhd2 over-expressing strains, respectively. We purified total RNA from cells collected 6 h after DS and tested the mRNA level of *SG4, IDP2* and *ODC1*. The results (Figure 3) show a consistent (around 50%) reduction in the induction level of all three genes in the ΔJHD2 strain and a very low expression in the Jhd2 over-expressing strain, which shows a 20-fold increase of *JHD2* mRNA at DS (Figure S3). These results confirm the hypothesis that an increase in the level of H3K4 tri-methylation at the proximal portion of the genes’ coding region is required for an efficient induction at DS. The stronger effect observed upon Jhd2 over-expression would suggest that in this case an active demethylation of a larger chromatin domain could be involved. 

**Figure 4:**
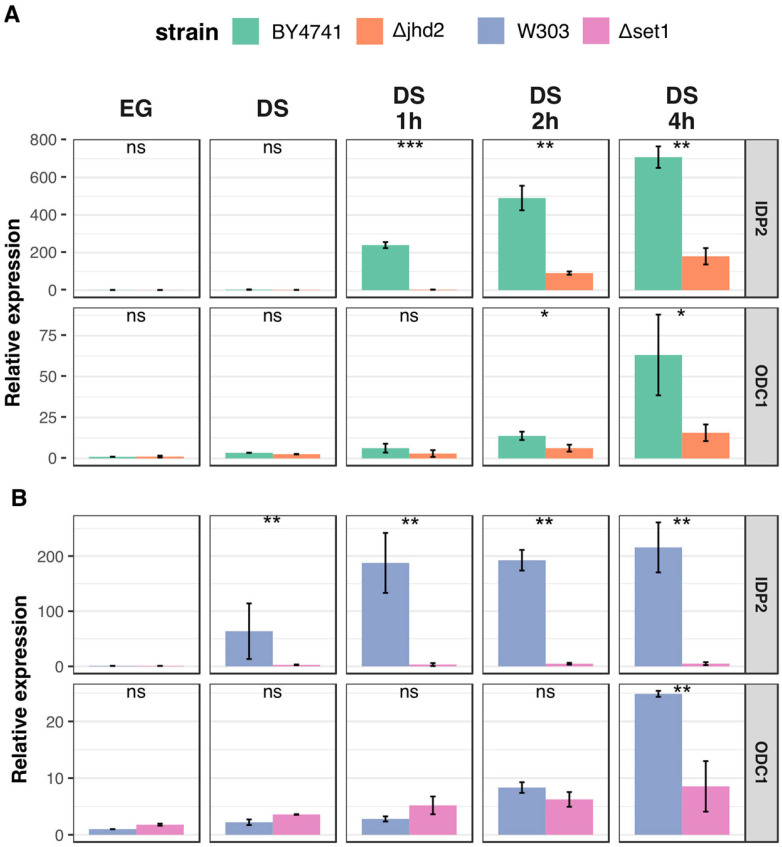
(**A**) Real time RT-PCR quantitation of the time course of induction of IDP2 and ODC1 in the W303 wild type and ΔSET1 strains. Data are the average of three independent experiments. Standard deviation is indicated. ns = not significant, * = *p*-value < 0.05; ** = *p*-value < 0.01; ***= *p*-value < 0.001 according to Student *t*-test. (**B**) Real time RT-PCR quantitation of the time course of induction of IDP2 and ODC1 in the BY4741 wild type and ΔJHD2 strains. Data are the average of three independent experiments. Standard deviation is indicated. * = *p* < 0.05; ** = *p* < 0.01; *** = *p* < 0.001 according to Student’s *t*-test.

We also tested the kinetics of induction in the ΔSET1and in the ΔJHD2strains as compared with their isogenic wild type strains. Samples were taken during exponential growth, at DS and at different times after DS. Figure 4 shows data obtained by real time RT-PCR for *IDP2* and *ODC1* genes. Strikingly, the induction of *IDP2* is strongly reduced also in the ΔJHD2strain, coherently with the lack of H3K4 tri-methylation observed. For *ODC1* the reduction of transcriptional induction is less evident (Figure 4A). Results show that both genes are poorly induced in the ΔSET1 strain as compared with the wild type (Figure 4B).

**Figure 5:**
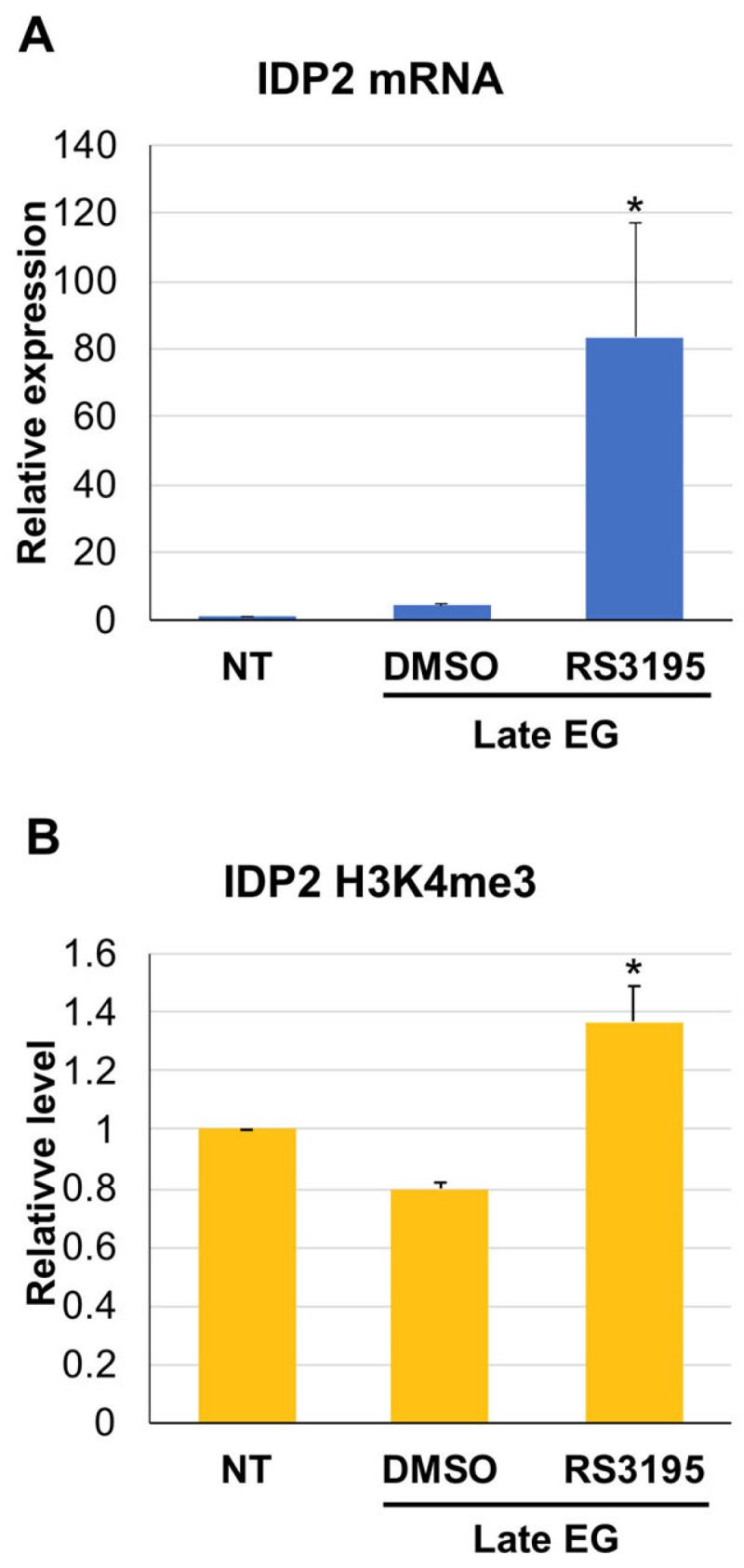
(**A**) Real time RT-PCR quantitation of IDP2 cDNA 2 h after addition of 15 μM RS3195 or the solvent 1% DMSO. The histogram reports the expression level normalized to the endogenous calibrator (*ACT1* mRNA) and to the untreated sample (ΔΔCt). Data are the average of three independent experiments. Late EG = 0.8–1 OD 600. (**B**) Real time quantification of ChIP experiments determining the level of H3K4 trimethylation of *IDP2* proximal ORF portion in the presence of the Jhd2 inhibitor RS3195. The histogram represents the H3K4me3 level of IDP2 in the presence of 15 μM RS3195 or 1% DMSO, normalized to the endogenous calibrator (*ZRT1* ORF) and to the untreated sample (ΔΔCt). Data are the average of three independent experiments. Standard deviation is indicated. * = *p* < 0.05 according to Student’s *t*-test, two sided, in comparison to NT.

Since experiments with genetic variants could be misleading (i.e., the ΔSET1 strain grows much slower than the wild type and has a different metabolism; Set1 has H3-K4 methylation-independent functions) we tested the effect of H3K4 tri-methylation on *IDP2* induction making use of the small molecule RS3195 which has been previously shown to inhibit the catalytic activity of Jhd2 in yeast and of its orthologues Jarid HDMs in mammalian cells [23,24]. We grew wild type cells to late exponential phase and split the culture in two aliquots: one treated with DMSO (solvent of RS3195) and the other with the RS3195 inhibitor. Then we collected cells after 2 h and purified total RNA. Figure 5A shows that the Jhd2 demethylase inhibitor promotes an anticipated induction of *IDP2*. Control ChIPs show a slight but significant increase of H3K4 tri-methylation in the *IDP2* ORFs (Figure 5B). A similar trend, although not statistically significant due to high variability, is observed for *ODC1* (Figure S4). So, in the case of a wild type strain in which both the methylase and the demethylase activities are present, perturbing their dynamic equilibrium toward an early methylation increase leads to an anticipated transcriptional induction. Something that does not happen when the demethylase activity is absent from the beginning.

### 3.4. Active Demethylation Is Not Required for DS Genes Repression following Refreshing

Since we showed that H3K4 tri-methylation is required for full activation of several DS induced genes, we tested if demethylation was required for their repression following addition of fresh medium with glucose. We grew WT or ΔJHD2strains and collect cells in exponential growth and two hours after the growth plateau. Then we resuspended part of cells in fresh medium with glucose and took samples at 30′, 1 and 2 h. Figure S5 shows that although *IDP2* is less induced after DS in the ΔJHD2strain, it is promptly repressed as the wild type, ruling out a role for the Jhd2 demethylase in repression.

## 4. Discussion

This work started with the observations that both genes coding for the H3K4 methylase Set1 and for the demethylase Jhd2 are induced before and expressed during DS and that the Jhd2 protein is present at DS and accumulates immediately after DS exit. This suggests a role for H3K4 methylation in regulating gene expression during DS. Indeed, we observed an evident increase of H3K4 tri-methylation in two different wild type genetic contexts when the cells enter DS. Thus, we performed a genome-wide ChIP on chip screening to identify genomic loci which show a H3K4 tri-methylation increase of at least 20 percentile ranks. We identified 86 ORF and 85 intergenic regions. 45 of the 86 ORF corresponded to genes highly transcriptionally induced in DS. Most of them code for proteins involved in the change of cellular metabolism at DS. To understand if H3K4 tri-methylation was a consequence of transcriptional induction of these genes or played a role in their activation, we analysed the effects of perturbing the methylation balance by genetic or pharmacological approaches.

**Figure 6:**
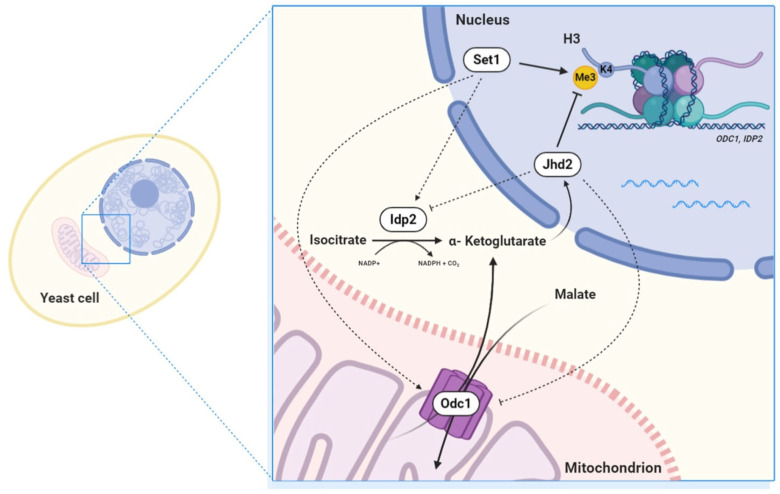
Suggested scenario for a feedback regulation linking IDP2 and ODC1 expression to α-Ketoglutarate availability in the nucleus.

We focused on two genes in particular: *IDP2* and *ODC1*. The *IDP2* gene codes for the cytosolic NADP-specific isocitrate dehydrogenase that catalyses oxidation of isocitrate to α-ketoglutarate [20]. Its levels are elevated during growth on non-fermentable carbon sources and reduced during growth on glucose and the gene is strongly induced at DS. Odc1 is a mitochondrial inner membrane transporter which transports 2-ketoglutarate from the mitochondrion to the cytoplasm [21]. The two proteins have a key role in regulating the nuclear α-ketoglutarate’s pool which in turn is the Jhd2 demethylase cofactor. It has been shown that α-ketoglutarate concentration is important in determining chromatin transcriptional state [25]. Moreover, recent studies revealed that oncogenic mutations in human IDH1/2 genes result in the synthesis of 2-hydroyglutarate (2-HG) instead of the normal α-ketoglutarate product and that 2-HG competitively inhibits JMJC-domain histone demethylases, resulting in increased H3K9 methylation [26,27]. Crosstalk between histone demethylation and hypoxic reprogramming is crucial in cancer metabolism [28,29]. Further, levels of α-ketoglutarate are key for transcriptional and epigenetic processes in stem cell maintenance [30]. Recent work by Meneghini’s group demonstrated that Jhd2′s role in demethylation and transcription regulation becomes prominent in yeast fermenting cells manipulated to contain an elevated α-ketoglutarate/succinate ratio and in respiratory cells [31]. It is therefore expected that genes controlling α-ketoglutarate availability in the nucleus may be subjected to a feedback control by methylating/demethylating enzymes. We show here that H3K4 tri-methylation contributes to the transcriptional induction of both *IDP2* and *ODC1*. In particular: mRNA levels of *IDP2* can be prematurely increased in late exponential growth phase by inhibiting the catalytic activity of Jhd2 demethylase and both *IDP2* and *ODC1* activation is impaired in strains deleted in *SET1* or over-expressing Jhd2. Based on this observation we propose a scenario in which the balance between H3K4 methylase and demethylase activities in DS cells regulates the expression of genes which control the available concentration of α-ketoglutarate in the nucleus, which in turn regulates the demethylase activity in a feedback loop (Figure 6). This is a further example of the metabolic epigenetic regulation which seems to be very relevant in several eukaryotic biological systems. HMT and HDMs seem to be very sensitive to availability of SAM and α-ketoglutarate, respectively, and H3K4 methyl state is partly determined by their equilibrium [32]. Metabolic control of histone methylation in mammalian cells has profound implication on cancer development [33]. Several crosstalk circuits between epigenetics and metabolism involving HDMs have been described in human cells [34]. For what regards yeast, recent work [10] showed that for most of the genes regulated by H3K4 methylation during exponential growth a combined action of Set1 and Jhd2 is required both for transcriptional induction and repression supporting the relevance of a dynamic equilibrium of H3K4 methyl state for controlling gene regulation. Another striking example is the glycolytic regulation of gene expression based on the crosstalk between H3K4 trimethylation and H3K14 acetylation [35].

In the course of this work, we found an interesting case of epigenetic adaptation: the increase of H3K4 tri-methylation observed in WT strains at DS is not observed in a strain deleted for Jhd2 demethylase. Indeed, the local increase of H3K4 tri-methylation at DS induced genes is not observed in this strain with consequent reduction in the extent of transcriptional induction. In search of a possible mechanism for this adaptation we considered the possibility of a feedback regulation of the abundance of Set1 protein, as recently observed [36]. On the contrary when we compared total cell extracts from ΔJHD2 strain with an isogenic WT strain we found similar quantities of Set1 both in exponential growth and after DS (Figure S6). We could therefore speculate that the observed reduction of H3K4 tri-methylation is due to a decreased recruitment of the Set1 methylase at the specific transcription units in the absence of Jhd2. A local regulatory crosstalk between the two enzymes has been previously suggested [10]. Recent work shows tight coordination of Set1 complex and Jhd2 action, especially at promoters of genes involved in the oxidative phase of the metabolic cycle [37]. Further work will be required to understand which mechanism is involved in this adaptation.

In conclusion H3K4 tri-methylation appears to have an important role for the induction of several genes involved in the metabolic changes required during DS. Other mechanism besides the simple balance between methylase and demethylase activities were recently shown to operate in defining the final methylation state of this category of genes [37,38] which could contribute to their regulation, but we think that the α-ketoglutarate feedback circuit which we described can be an important piece of the puzzle.

## Figures and Tables

**Table 1:** Yeast strain used.

Yeast Strain	Genotype
BY4741	*MATα his3∆ leu2∆0 LYS2 met15∆0 ura3∆0*
W303	*MATa, his3-11, ade2-1, leu2-3,112, ura3-1, trp1-D2, can1-100*
Δjhd2 BY4741	*MATa, his3∆ leu2∆0 LYS2 met15∆0 ura3∆0 YJR119c::kanMX4*
Δset1 W303	*MATa, his3∆ leu2∆0 LYS2 met15∆0 ura3∆0 YJHR119w::kanMX4*
over-JHD2	as BY4741 with plasmid pDPM2

**Table 2:** Genes involved in the change of metabolism imposed by DS.

Gene	Description	Rank Difference (DS-EG)	*p*
IDP2	Isocitrate dehydrogenase (NADP+), cytosolic	0.78	<0.001
MLS1	Malate synthase 1, functions in glyoxylate cycle, has near identity to Dal7p	0.57	<0.01
ECM13	Protein possibly involved in cell wall structure or biosynthesis	0.55	<0.01
GND2	6-Phosphogluconate dehydrogenase, decarboxylating, converts 6-phosphogluconate + NADP to ribulose-5-phosphate + NADPH + CO_2_	0.49	<0.001
PXA1	Protein required for long-chain fatty acid transport across the peroxisomal membrane, member of the ATP-binding cassette (ABC) superfamily, has similarity to a human gene associated with adrenoleukodystrophy	0.49	<0.001
YPT53	GTP-binding protein involved in endocytosis and transport of proteins to the vacuole, member of the rab family in the ras superfamily	0.48	<0.01
ODC1	2-Oxodicarboxylate transporter, has specificity for 2-oxoadipate and 2-oxoglutarate, member of the mitochondrial carrier (MCF) family of membrane transporters	0.48	<0.05
ATH1	Vacuolar acid trehalase, converts alpha, alpha-trehalose to glucose	0.47	<0.001
GPX1	Glutathione peroxidase, involved in cellular protection against lipid and non-lipid hydroperoxides	0.45	<0.05
MTH1	Repressor of hexose transport genes	0.45	<0.05
ADY2	Protein required for proper ascus formation, has strong similarity to Ydr384p and Ynr002p	0.44	<0.05
HSP26	Heat shock protein of 26 kDa, expressed during entry to stationary phase and induced by osmostress, may be required for resistance to ethanol and acetaldehyde	0.42	<0.001
LEE1	Protein containing two CCCH-type zinc finger domains, which bind DNA or RNA	0.4	<0.001
TRX3	Mitochondrial thioredoxin, has similarity to cytoplasmic thioredoxins Trx1p and Trx2p	0.38	<0.05
CRC1	Mitochondrial carnitine carrier, member of the mitochondrial carrier family (MCF) of membrane transporters	0.38	<0.001

## Data Availability

ChIP on chip data is provided in Table S3.

## Supplementary Materials

The following supporting information can be downloaded at: https://www.mdpi.com/article/10.3390/metabo13040507/s1, Figure S1: JHD2, IDP2 and SET1 levels during various phases of growth; Figure S2: ChIP analysis of H3K4 trimethylation of HAS1 promoter in the wild type BY4741 and isogenic Djhd2 strain; Figure S3: Real time RT-PCR analysis of JHD2 mRNA accumulation; Figure S4: Catalytical inhibition of Jhd2 causes an increase of H3K4 trimethylation and an early transcriptional induction of ODC1; Figure S5: Time course of repression of IDP2 and ODC1 after release from DS for the wild type BY4741 and the isogenic ΔJHD2 strain; Figure S6: Set1 protein levels in Jhd2 absence; Table S1: RT-PCR primers sequences; Table S2: selected list of ChIP on chip results. Reference [19] is cited in Supplementary Materials.
