# Supplementary material for: H3 Lysine 4 Methylation Is Required for Full Activation of Genes Involved in α-Ketoglutarate Availability in the Nucleus of Yeast Cells after Diauxic Shift
Source: Metabolites. 2023 Mar 31;13(4):507. doi: 10.3390/metabo13040507 (PMC10146420; doi:10.3390/metabo13040507)
Supplement: Supplementary file 1 [file metabolites-13-00507-s001.zip › metabolites-2282252-supplementary.pdf]

Supplementary Figures

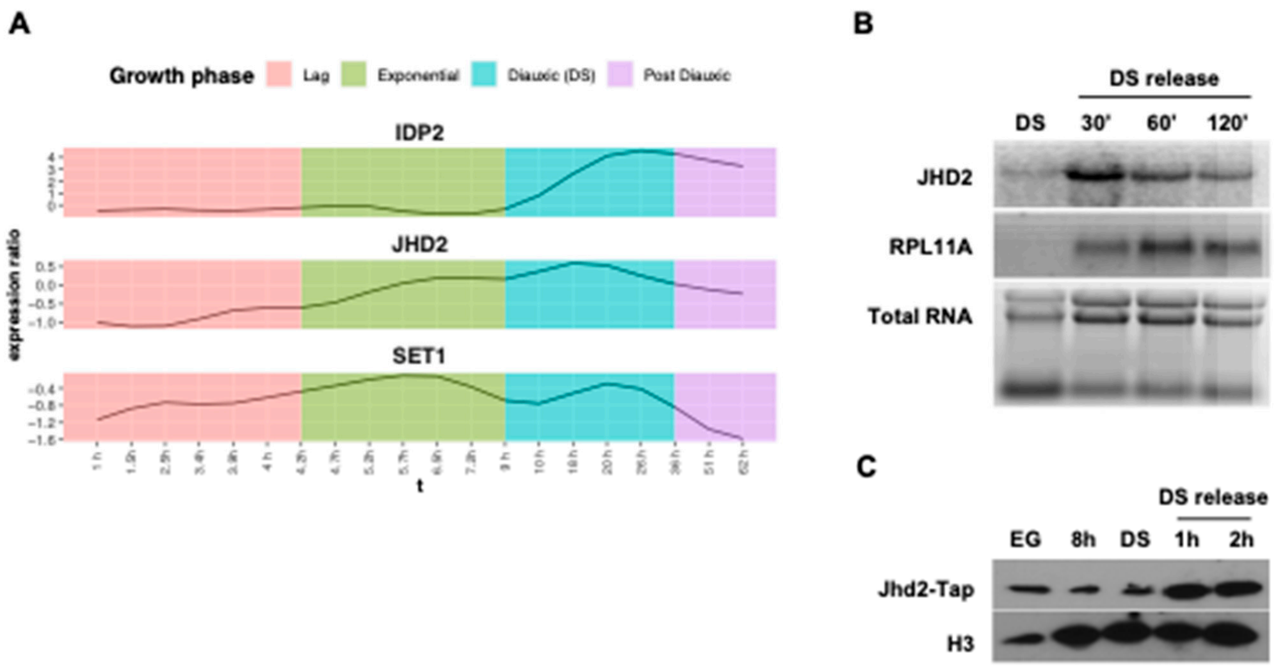

Figure S1. JHD2, IDP2 and SET1 levels during various phases of growth. A, Expression of IDP2, SET1 and JHD2 mRNA during various phases of growth. Data from [19]. **B**, Northern blot showing JHD2 (top) and RPL11A (middle) mRNA at DS and at the indicated time points after addition of fresh medium plus glucose. Total RNA from the same gel is shown at the bottom. **C**, Western blot showing the Jhd2-TAP fusion protein in the first phase of exponential growth (EG) and after 8 hours; at Diauxic shift phase and at the indicated times after addition of fresh medium plus glucose. Histone H3 is used as loading control.

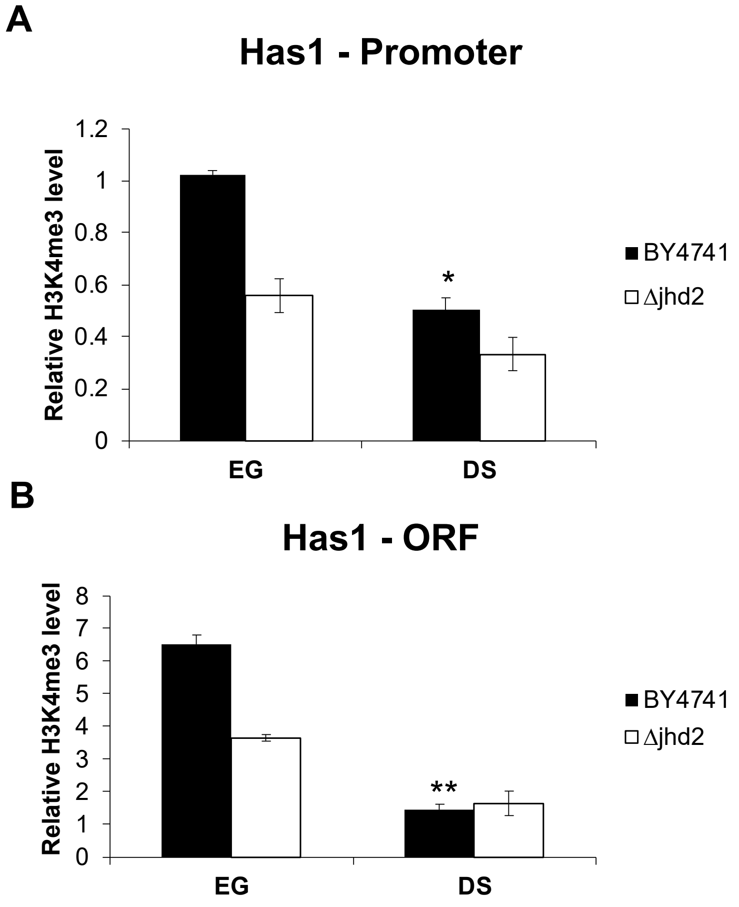

Figure S2. ChIP analysis of H3K4 trimethylation of HAS1 promoter in the wild type BY4741 and isogenic Djhd2 strain. Data are the average of three independent experiments. Standard deviation is indicated. * = $p < 0.05$; ** = $p < 0.01$ according to Student's t-test.

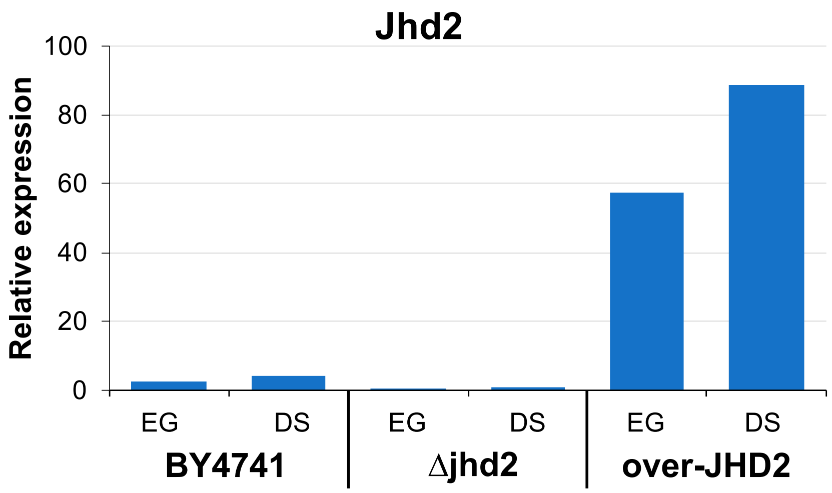

Figure S3. Real time RT-PCR analysis of JHD2 mRNA accumulation in WT, Djhd2 and over-JHD2 strains at exponential growth (EG) and diauxic shift (DS). Typical experimental result is shown.

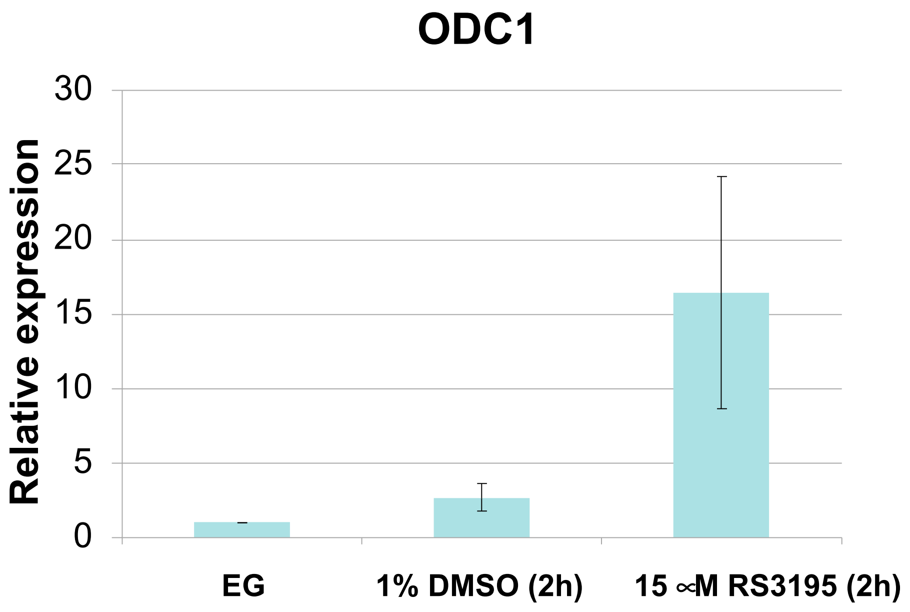

Figure S4. Catalytical inhibition of Jhd2 causes an increase of H3K4 trimethylation and an early transcriptional induction of ODC1. Quantification by Real time RT-PCR of ODC1 cDNA 2h after addition of 15 mM RS3195 or the solvent 1% DMSO. The histogram reports the expression level normalized to the endogenous calibrator (ACT1) and to the untreated sample (ΔΔCT). Data are the average of three independent experiments. Standard deviation is indicated. Although treatment with the inhibitor increases ODC1 expression in late EG, data are not statistically significant due to high variability. Late EG = 0.8-1 OD_{600}

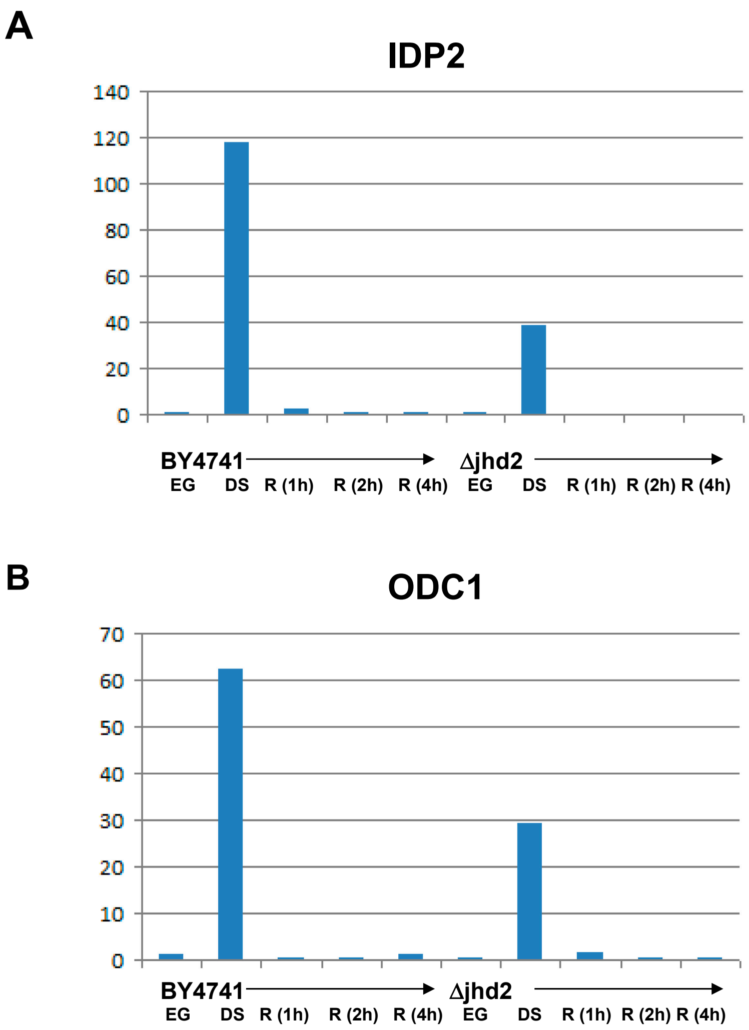

Figure S5. Time course of repression of IDP2 and ODC1 after release from DS for the wild type BY4741 and the isogenic DJHD2 strain. A, Quantification by real time RT-PCR for IDP2 (**A**) and for ODC1 mRNAs (**B)**. Data represent the expression normalized to the endogenous calibrator TAF10 and relative to the samples in EG.

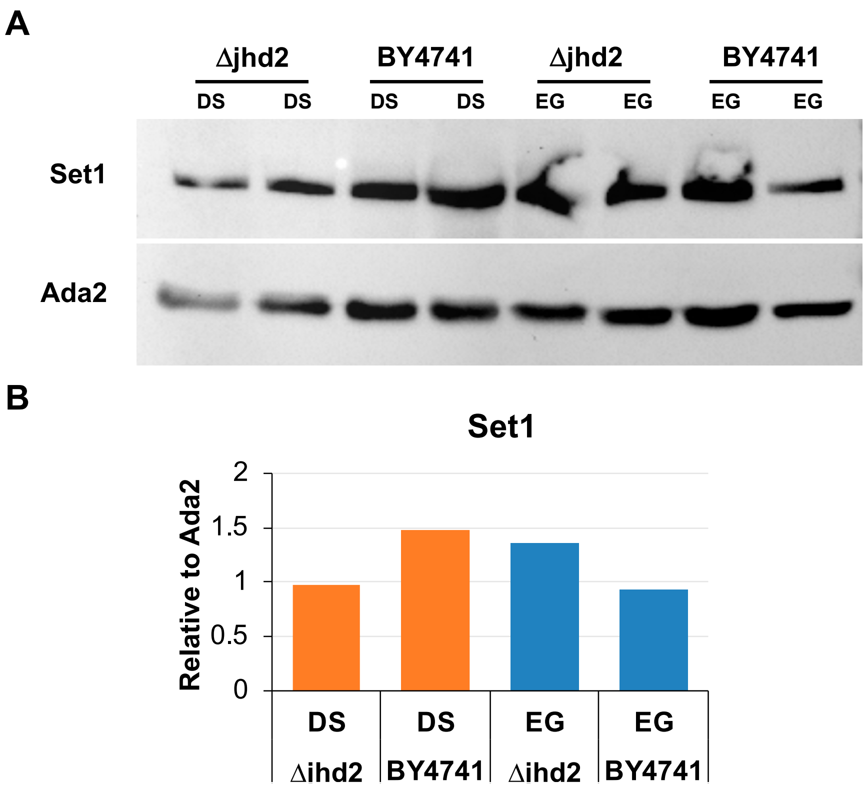

Figure S6. Set1 protein levels in Jhd2 absence. A, Western blot showing the abundance (n = 2) of Set1 in EG and DS in the wild type BY4741 and in the isogenic Djhd2 strains. **B**, Average Ratio between the Set1 and the endogenous calibrator Ada2 bands densitometric values are reported for each condition and strain.
